# A new method for estimating three-dimensional movement of the patella using a surface mapping method and computed tomography

**DOI:** 10.1016/j.heliyon.2020.e04729

**Published:** 2020-08-18

**Authors:** Takuma Inai, Tomoya Takabayashi, Satoshi Watanabe, Masahiro Ikezu, Fumiya Kaneko, Kanta Matsuzawa, Mutsuaki Edama

**Affiliations:** a Institute for Human Movement and Medical Sciences, Niigata University of Health and Welfare, Niigata, Japan; b Department of Rehabilitation, Niigata Medical Center, Niigata, Japan

**Keywords:** Biomedical engineering, Computing methodology, Computer simulation, Bioengineering, Biomechanics, Biomechanical engineering, Patella, Surface mapping method, Computed tomography, Kinematics, Knee

## Abstract

**Introduction:**

A previous study reported a method called the 2D-3D registration technique to examine three-dimensional movement of the patella. However, that method requires a biplane fluoroscopy system. In the present study, the aim was to establish a new method (CT-based surface mapping method) to estimate three-dimensional positions and angles of the patella with a motion capture system and CT.

**Methods:**

In Study 1, the most appropriate parameters for the CT-based surface mapping method (i.e., target edge length, threshold of thickness of the soft tissue, and minimum distance between markers) were explored and determined. In Study 2, three-dimensional movement (i.e., positions and angles) of the patella using the CT-based surface mapping method and the most appropriate parameters were determined, and they were compared with the true positions and angles obtained by CT.

**Results:**

The results of Study 1 showed that the most appropriate conditions were as follows: (1) target edge length, 3 mm; (2) threshold of thickness of the soft tissue, 0–20 mm; and (3) minimum distance between markers, 10 mm. The results of Study 2 showed that the errors of the positions and angles were less than approximately 10 mm and 10° at most, respectively (both supine and sitting positions).

**Conclusion:**

The CT-based surface mapping method may be useful for a future study to clarify differences in three-dimensional movements of the patella between patients with patellar tendinitis and healthy subjects.

## 1 Introduction

Patellar tendinitis (i.e., jumper's knee) is a sports injury that occurs in sports including jumping movements [1] and causes pain in the proximal patellar tendon [2]. A previous study reported that a decrease in flexibility of the quadriceps (i.e., decreased range of joint motion) increases the incidence of patellar tendinitis after two years [3], and another previous study reported that passive force of the vastus lateralis was significantly higher in a control group than in patients with patellar tendinitis [4]. Therefore, excessive muscle force of the quadriceps may cause “abnormal patellar tracking” based on these previous studies [3, 4], and the abnormal patellar tracking may cause biomechanical stress to the patellar tendon (i.e., repetitive tensile overload or impingement [5]). For these reasons, it is important to establish and develop a method to measure three-dimensional movement of the patella (i.e., positions and angles) (see Table 1).

**Table 1:** Total score in each condition (Study 1).

Condition			Total score
Target edge length [mm]	Thickness of the soft tissue [mm]	Minimum virtual marker distance [mm]	
2	0–5	10	312
2	0–5	15	344
2	0–5	20	439
2	0–12.5	10	245
2	0–12.5	15	326
2	0–12.5	20	375
2	0–20	10	257
2	0–20	15	273
2	0–20	20	327
2	0–5	10	394
3	0–5	15	397
3	0–5	20	470
3	0–12.5	10	297
3	0–12.5	15	348
3	0–12.5	20	314
**3**	**0–20**	**10**	**235**
3	0–20	15	269
3	0–20	20	261
4	0–5	10	374
4	0–5	15	410
4	0–5	20	384
4	0–12.5	10	347
4	0–12.5	15	409
4	0–12.5	20	353
4	0–20	10	251
4	0–20	15	331
4	0–20	20	330

The bold letters indicate the most appropriate condition.

Several previous studies that examined three-dimensional movement of the patella during human movements have been reported [6, 7, 8, 9]. These previous studies created three-dimensional patella models using magnetic resonance imaging (MRI) [7, 9] or computed tomography (CT) [6, 8], and they measured three-dimensional positions and angles of the patella using a biplane fluoroscopy system and the 2D-3D registration technique. However, the 2D-3D registration technique for the patella needs a biplane fluoroscopy system, and there are not many institutions (e.g., hospitals and research institutions) that have biplane fluoroscopy systems. Therefore, it is important to establish a new method to measure three-dimensional movement of the patella using other experimental systems.

Mattson et al. (2012) examined three-dimensional movement of the scapula using the surface mapping method. It may be possible to estimate the movement of the patella using this method. However, they created a three-dimensional bone model of the scapula by palpation. The accurate shape of a three-dimensional bone model cannot be created based on palpation. Therefore, we created an accurate three-dimensional bone model of the patella using CT and combined the bone model and the surface mapping method. We call this new method the "CT-based surface mapping method". With this method, it may be possible to conduct a static evaluation of the patella with a three-dimensional motion capture system and CT (or MRI).

To perform CT-based surface mapping, several parameters (target edge length, threshold of thickness of the soft tissue, and distance between markers on skin) are needed. Therefore, the first purpose of this study was to explore and determine the most appropriate parameters (target edge length, threshold of thickness of the soft tissue, and distance between markers on skin) for the CT-based surface mapping method (Study 1). Furthermore, in Study 2, three-dimensional movement of the patella was examined using the CT-based surface mapping method and the most appropriate parameters. The second purpose of this study was to compare the three-dimensional movement of the patella using the CT-based surface mapping method and the true degrees of freedom (positions and angles) of the patella obtained by CT data (Study 2).

## 2 Materials and methods

As stated in the purpose, two studies were performed. Three parameters (target edge length, threshold of thickness of the soft tissue, and minimum distance between markers on the skin) are needed for the CT-based surface mapping method. The most appropriate parameters were explored and determined in Study 1, and the most appropriate parameters were then used for Study 2.

**Figure 1:**
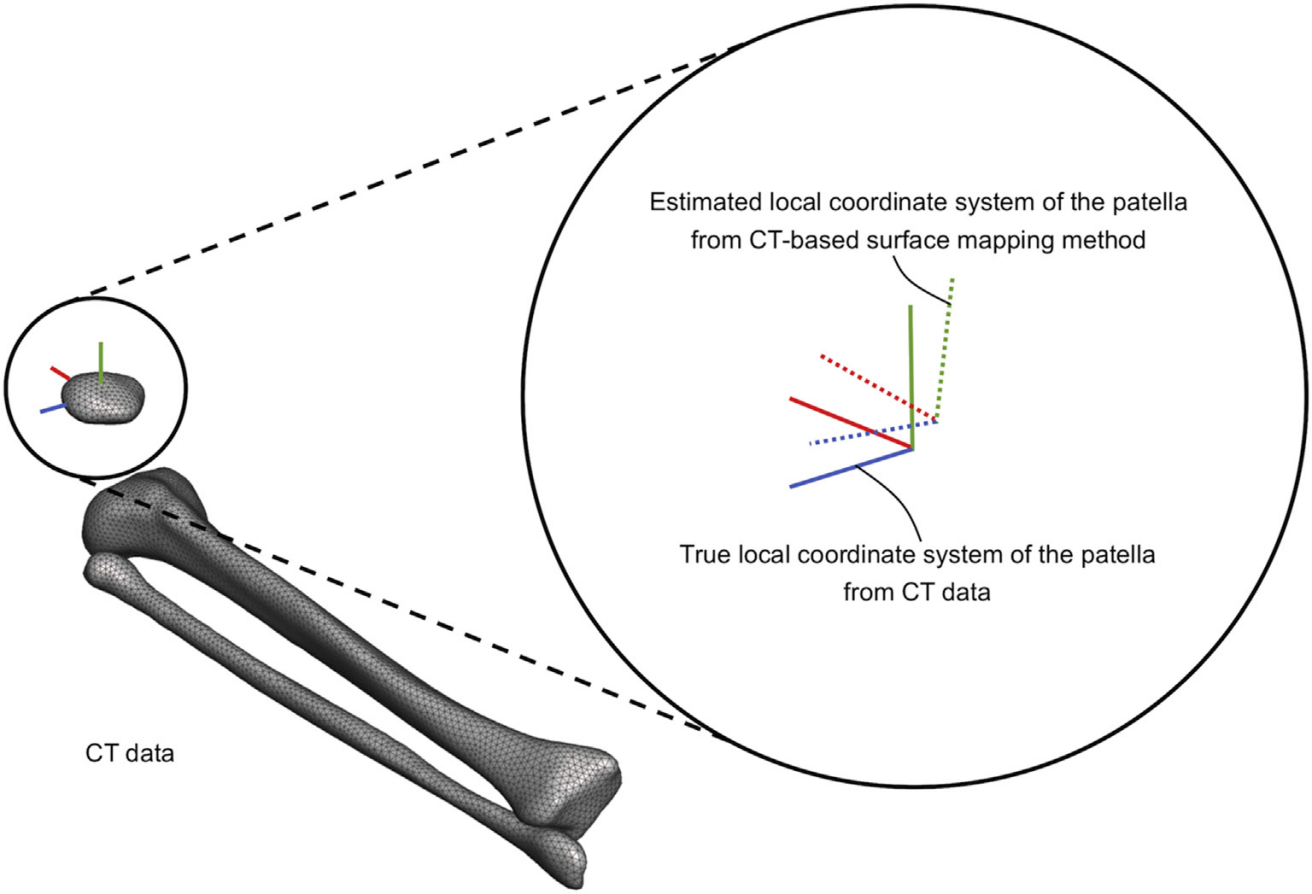
True and estimated local coordinate systems of the patella. The true local coordinate system of the patella is obtained from CT data (solid line). The estimated local coordinate system of the patella is obtained from the CT-based surface mapping method (dotted line).

In both studies, three types of local coordinate systems of the patella were needed: (1) the true local coordinate system of the patella (i.e., the local coordinate system obtained from CT); (2) the initial local coordinate system of the patella (i.e., the local coordinate system used for the CT-based surface mapping method); and (3) the estimated local coordinate system of the patella (i.e., the local coordinate system obtained from the CT-based surface mapping method). Figure 1 shows the details of the local coordinate systems. The initial local coordinate system of the patella was used as an initial position of the local coordinate system of the patella in the CT-based surface mapping method. Displacements (i.e., errors) of the origin (x, y, and z axes) and cardan angles (x, y, and z axes) between the true and estimated local coordinate systems of the patella were calculated and compared in both studies.

### 2.1 Study 1

**Figure 2:**
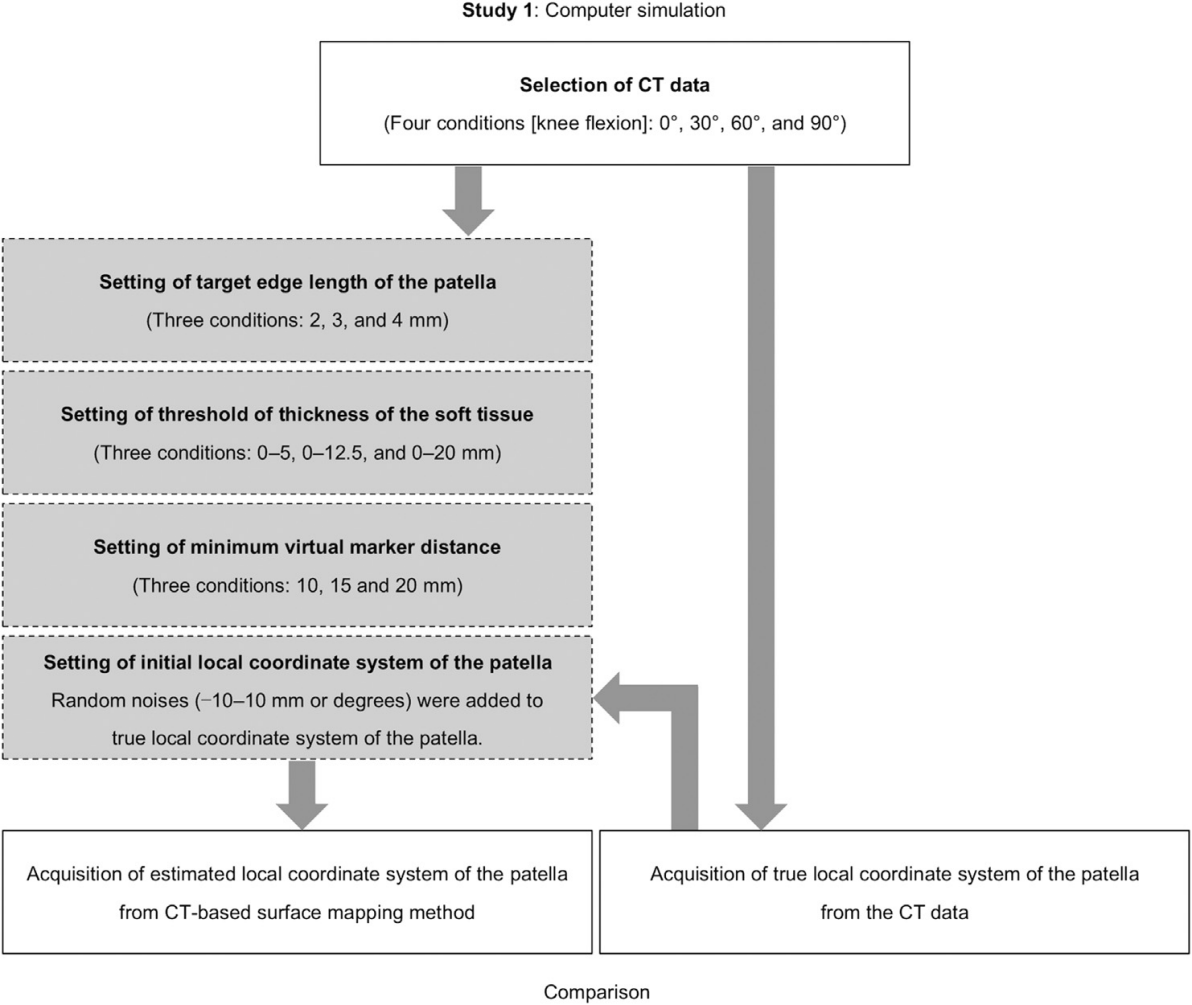
Flowchart of Study 1. The left column indicates the process of the CT-based surface mapping method in Study 1. The right column indicates the process to obtain the true local coordinate system of the patella from CT data.

Figure 2 shows the flowchart of Study 1. The purpose of Study 1 was to explore and determine the most appropriate parameters for the CT-based surface mapping method (i.e., target edge length, threshold of thickness of the soft tissue, and minimum distance between markers).

#### 2.1.1 Subject

A healthy 23-year-old man participated in Study 1 (height: 1.68 m, body mass: 61.0 kg). Inclusion criteria were: no pain in the knee joint; no disease in the knee joint (e.g., knee osteoarthritis); and no history of surgery of the knee joint. This study was approved by the institutional review board of Niigata University of Health and Welfare (Approved number: 18271). Written, informed consent was obtained from the subject before participation.

#### 2.1.2 Collection of CT data

The right lower limb from the right foot to the thigh of the subject was scanned by CT (Aquilion TSX-101A, Canon Medical Systems Inc, Tochigi, Japan). The subject was asked to be in the supine position under four conditions, as follows: (1) 0° knee flexion; (2) 30° knee flexion; (3) 60° knee flexion; and (4) 90° knee flexion. The knee joint angle was adjusted using a goniometer before scanning the lower limb by CT. Appendix A explains the creation of the three-dimensional model and definition of the local coordinate system of the patella.

#### 2.1.3 Setting of true and initial local coordinate systems of the patella

The true local coordinate system of the patella was obtained from CT data (details in Appendix A). The initial local coordinate system of the patella was generated by adding random positions and angle values (±10 mm or degrees) to the true local coordinate system of the patella to examine whether appropriate solutions can be obtained even if the initial values have errors with respect to the true solutions.

#### 2.1.4 Setting of target edge length, threshold of thickness of the soft tissue, and minimum distance between virtual markers

**Figure 3:**
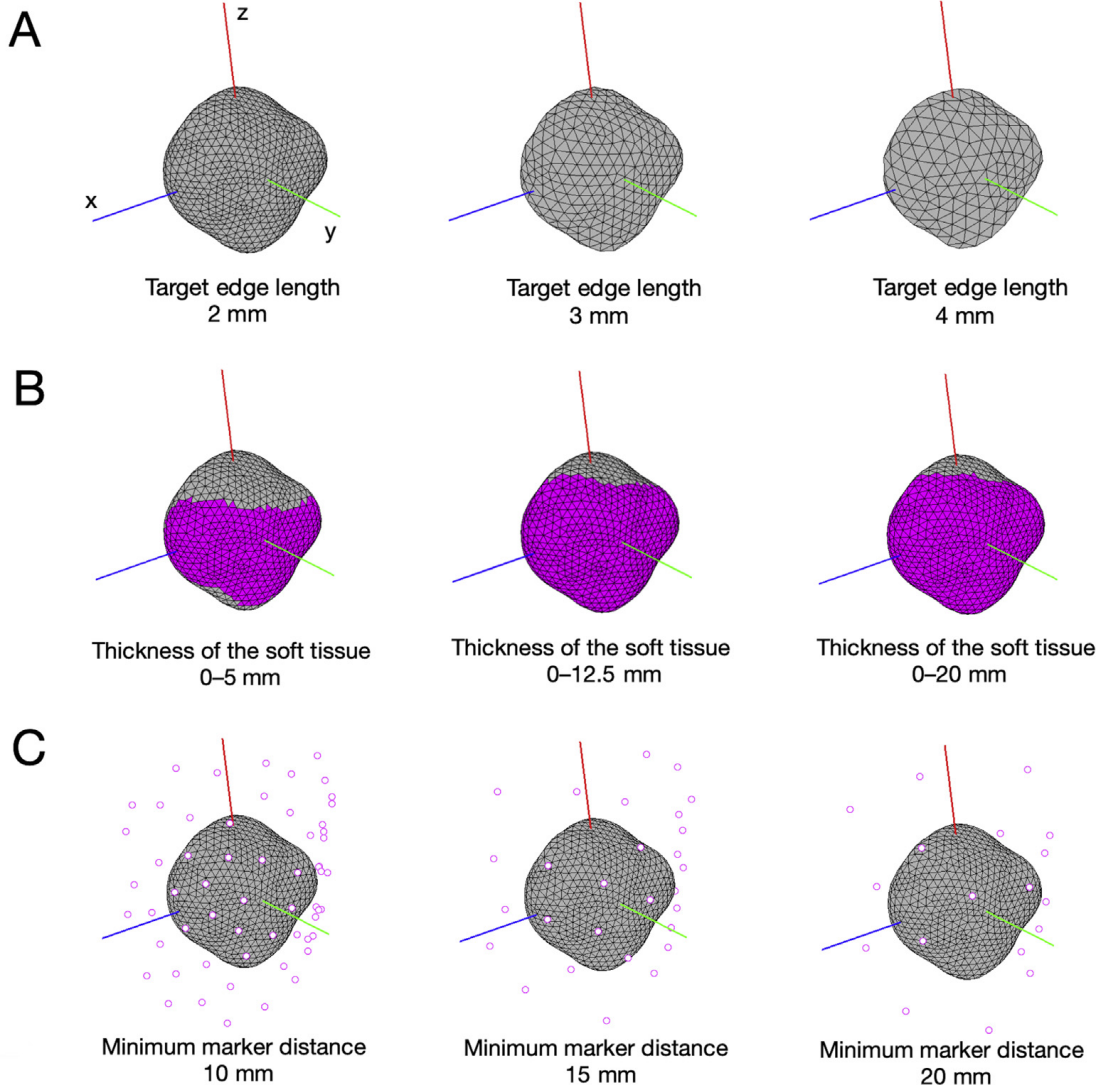
Various conditions for Study 1. A: Three conditions (2, 3, and 4 mm) are set for the target edge. B: Three conditions (0–5, 0–12.5, and 0–20 mm) are set for thickness of the soft tissue. C: Three conditions (10, 15, and 20 mm) are set for the minimum virtual marker distance.

Differences in target edge length may affect estimates of the three-dimensional positions and angles of the patella. Therefore, three conditions were set (Figure 3A): (1) Target edge length = 2 mm; (2) Target edge length = 3 mm; and (3) Target edge length = 4 mm. In addition, uniformity was set to 100% in Meshmixer (Autodesk, Inc., CA, USA). Note that one can only approximately satisfy a given edge-length criteria [11]. This is the nature of the remeshing algorithm; it is extremely difficult, mathematically, to find a mesh that has tightly bounded edge lengths. One can at best provide an approximation, with roughly 50% deviation from the target value. When target edge length is less than 2 mm, extremely high computational cost occurs. Further, when target edge length is greater than 4 mm, the bone model of the patella is too different from its original form. Therefore, the range was set from 2 mm to 4 mm.

In a previous study of the surface mapping method [10], several surfaces were created from markers on the skin, and the sum of the distances between the surfaces and the landmarks of the scapula was calculated. This calculation was modified in the present study, and the sum of the distances between markers on the skin and the surfaces of the triangle mesh of the patella was calculated. Therefore, the area of the triangle mesh of the patella needed to be adjusted for analysis, so vertical distances between each triangle mesh of the patella and skin surface were calculated and set to three thresholds (Figure 3B): (1) 0–5.0 mm; (2) 0–12.5 mm; and (3) 0–20.0 mm. The threshold of thickness of the soft tissue was such that only the triangle meshes of which the distances to the skin surface are within a certain value can be used for surface mapping. A greater value leads to more meshes involved in surface mapping; a lower value leads to fewer meshes. When the threshold of thickness of the soft tissue is less than 5 mm, the analysis area of the patella is too small, and the CT-based surface mapping method cannot be used. Furthermore, when the threshold of thickness of the soft tissue is greater than 20 mm, the analysis area of the patella is too large (e.g., there are some meshes for the analysis area in the posterior of the patella). Therefore, the range was set from 0–5 mm to 0–20 mm.

The shape of the patella is reflected by markers, and an accurate shape is necessary for determining degrees of freedom of the patella using the CT-based method. The distance between (virtual or experimental reflective) markers may also affect the accuracy of the positions and angles of the patella in the CT-based surface mapping method; therefore, three conditions of the minimum distance between virtual markers were set (Figure 3C): (1) 10 mm; (2) 15 mm; and (3) 20 mm. Using the coordinate points of the skin, virtual markers on the skin of the knee were generated using random numbers. When the distance between markers is less than 10 mm, the condition for Study 2 cannot be applied (i.e., actual reflective markers cannot be attached on the skin of the knee within a distance of 10 mm in an experiment). Furthermore, when the distance between markers is more than 20 mm, there are too few markers on the skin of the knee. Therefore, the range was set from 10 mm to 20 mm.

Four conditions of knee flexion angles (0°, 30°, 60°, and 90°), three conditions of the target edge length of the patella (2, 3, and 4 mm), three conditions of threshold of thickness of the tissue (0–5, 0–12.5, and 0–20 mm), and three conditions (10, 15, and 20 mm) of virtual marker distance were set. All combinations were simulated 10 times; therefore, 1080 simulations (i.e., = 3 × 3 × 3 x 4 × 10) were conducted.

#### 2.1.5 Analysis

Average errors for each degree of freedom for each condition (27 conditions) were calculated. Each degree of freedom of all conditions was scored (score of minimum error was 1, and score of maximum error was 27). The total score for each condition was calculated, and the parameters of the minimum total score were judged to be the most appropriate parameters. The detailed calculation processes are presented below in the “CT-based surface mapping method” section.

### 2.2 Study 2

**Figure 4:**
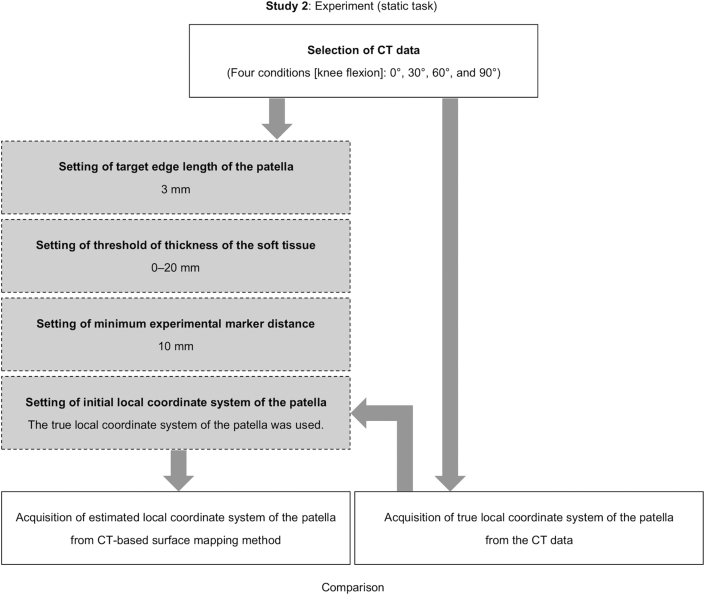
Flowchart of Study 2. The left column indicates the process of the CT-based surface mapping method in Study 2. The right column indicates the process to obtain the true local coordinate system of the patella from CT data.

Figure 4 shows the flowchart of Study 2. The second purpose of this study was to compare the three-dimensional movement of the patella using the CT-based surface mapping method and the true degrees of freedom (positions and angles) of the patella obtained by CT data (Study 2).

#### 2.2.1 Subjects

A 22-year-old healthy man participated in Study 2 (height: 1.63 m, body mass: 53.0 kg). The inclusion criteria were the same as Study 1. Written, informed consent was obtained from the subject before participation.

#### 2.2.2 Experiment using a motion capture system

Four conditions of the knee flexion angle (static task without muscle contraction) were set (0°, 30°, 60°, and 90°). Two conditions of posture were set (supine and sitting positions). Seventy-nine hemispheric reflective markers (diameter: 2 mm) were attached in a grid pattern on the anterior of the knee at 10-mm intervals. Moreover, four reflective markers (diameter: 20 mm) were attached on the medial condyle of the right tibia, the head of the right fibula, and the right medial and lateral malleoli. To capture these markers at all knee flexion angles, a motion capture system (Vicon, Oxford, United Kingdom) with eight cameras was used. Using these four reflective markers, the local coordinate system of the lower leg was created (see Appendix A for details).

#### 2.2.3 Collection of CT data

Similar to Study 1, the right lower limb from the right foot to the thigh of the subject was scanned by CT (Aquilion TSX-101A, Canon Medical Systems, Inc.). The subject was asked to be in the supine position under four conditions with the four reflective markers: (1) 0° knee flexion; (2) 30° knee flexion; (3) 60° knee flexion; and (4) 90° knee flexion. The knee joint angle was adjusted using a goniometer before scanning the lower limb by CT. In Study 2, four markers (diameter of 20 mm) were attached on the medial condyle of the right tibia, the head of the right fibula, and the right medial and lateral malleoli. Appendix A explains the creation of the three-dimensional model and definition of the local coordinate system of the patella and lower leg.

#### 2.2.4 Setting of true and initial local coordinate systems of the patella

Similar to Study 1, the local coordinate system of the patella obtained from CT data was used as the true local coordinate system of the patella, which was also used as the initial local coordinate system of the patella.

#### 2.2.5 Setting of target edge length, threshold of thickness of the soft tissue, and minimum distance between experimental markers

In Study 2, parameters obtained in Study 1 (target edge length: 3 mm, threshold of thickness of the tissue: 0–20 mm, and minimum distance between markers: 10 mm) were used (please see the Results section).

#### 2.2.6 Analysis

The estimated local coordinate system of the patella was calculated for each knee joint angle using the CT-based surface mapping method (see below section named “CT-based surface mapping method” for the detailed calculation processes). These estimated local coordinate systems of the patella were then compared to the true local coordinate system of the patella in each angle. Errors of positions and angles (Cardan angles) between them were calculated.

### 2.3 CT-based surface mapping method

**Figure 5:**
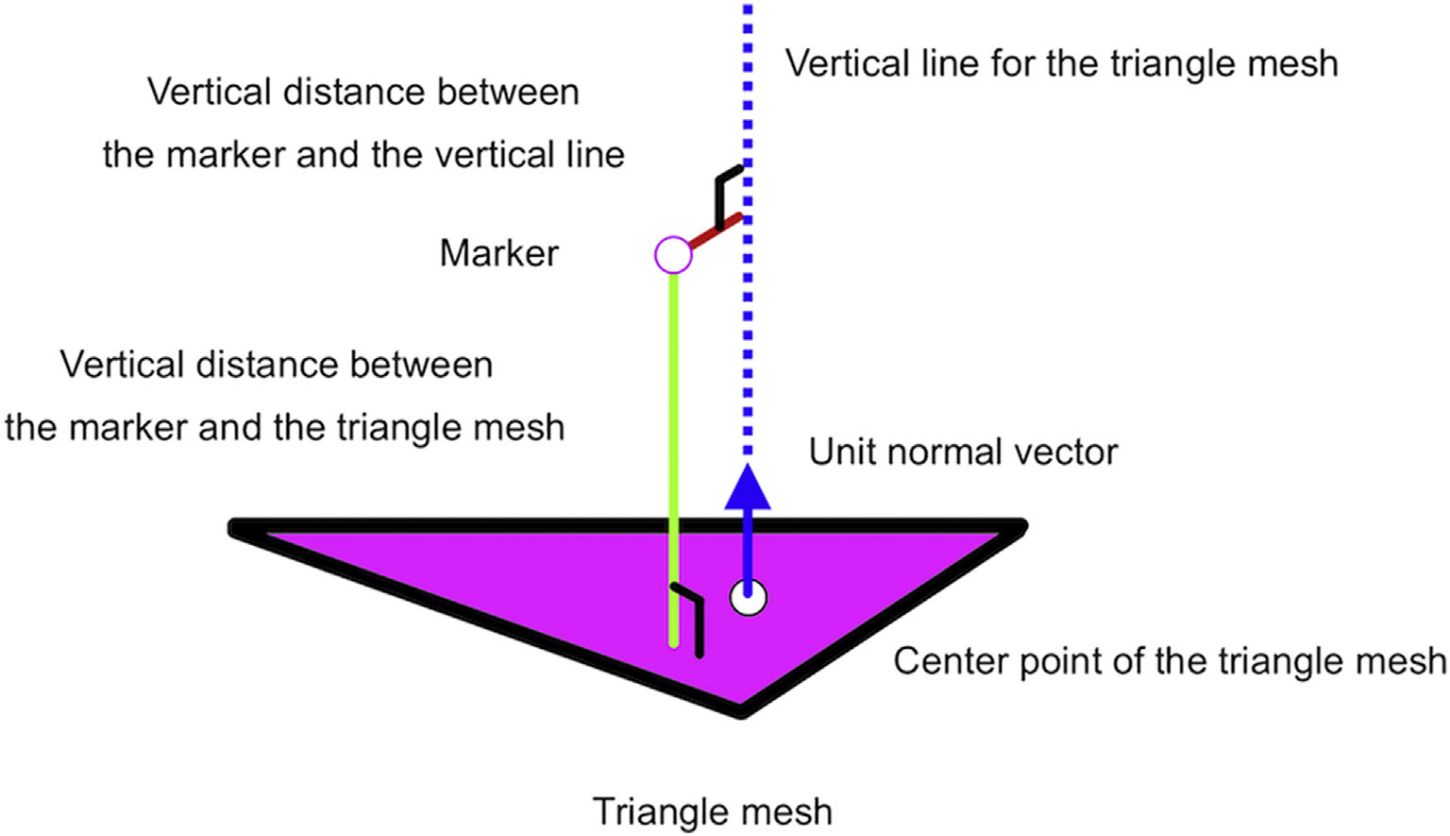
Process to calculate the vertical distance between the marker and the triangle mesh. The green line indicates the vertical distance between the marker and the triangle mesh.

Figure 5 shows the principle of the CT-based surface mapping method. First, in order to transform the 79 hemispheric reflective markers from the local coordinate system of the lower leg in the global coordinate system of a motion capture system to the local coordinate system of the lower leg in the global coordinate system of CT, the four markers attached on the lower leg were used.

Next, an analysis area out of all triangle meshes was defined based on the threshold of thickness of the soft tissue (i.e., magenta triangle meshes in Figure 3B). Then, a triangle mesh of the analysis area of the patella was extracted, and a unit normal vector was obtained on the triangle mesh. A marker (of 79 hemispheric reflective markers) closest to the vector was then identified, and the vertical distance between the marker and the triangle mesh was calculated. The procedure was applied for all triangle meshes in the analysis area.

When a surface mapping error Sconverges in the CT-based surface mapping method, the estimated local coordinate system of the patella is obtained. Additionally, the small surface mapping error S indicates that the patella fits the markers properly. The equation is as follows:

(1) $$Q=\left[\begin{array}{c} q_{1}\\ \vdots \\ q_{6} \end{array}\right]={\left[\begin{array}{cccccc} p_{\text{x}} & p_{\text{y}} & p_{\text{z}} & \theta_{\text{x}} & \theta_{\text{y}} & \theta_{\text{z}} \end{array}\right]}^{\text{T}}\text{,}$$

(2) $$D\left(Q,M\right)=\left[\begin{array}{c} d_{1}\\ \vdots \\ d_{N} \end{array}\right]\text{,}$$

(3) $$E=\left[\begin{array}{c} \varepsilon_{1}\\ \vdots \\ \varepsilon_{N} \end{array}\right]\text{,}$$

and

(4) $$S\left(Q,M,E\right)=\sum_{i=1}^{N}\left|d_{i}-\varepsilon_{i}\right|\text{,}$$

where Qis a matrix of the degrees of freedom of the patella, D is a matrix of the vertical distances between each triangle mesh of the analysis area and the corresponding marker, $d_{i}$is the vertical distance between the ith triangle and the corresponding marker, and N is the number of triangle meshes of the analysis area.$p_{\text{x}}$, $p_{\text{y}}$, and $p_{\text{z}}$ are positions (x, y, and z axes) of the origin of the local coordinate system of the patella. $\theta_{\text{x}},$$\theta_{\text{y}},$and $\theta_{\text{z}}$ are angles (x, y, and z axes) of the local coordinate system of the patella. $M\in R^{m\times 3}$is a matrix of (virtual [Study 1] or experimental [Study 2]) markers on the knee (m is the number of markers), E is a matrix of the vertical distances between each triangle mesh and the skin of the knee, $\varepsilon_{i}$is the vertical distance between the ith triangle mesh and the skin of the knee (i.e., the distance for each triangle mesh was calculated from CT data), and Sis the surface mapping error.

Moreover, to decrease surface mapping error S, the modified steepest descent method was used:

(5) $$H=\left[\begin{array}{ccc} h & \cdots & 0\\ \vdots & \ddots & \vdots \\ 0 & \cdots & h \end{array}\right]\text{,}$$

(6) $$S_{i,1}=S\left(Q,M,E\right)\text{,}$$

(7) $$S_{i,2}=S\left(Q+H_{i},M,E\right)\text{,}$$

(8) $$S_{i,3}=S\left(Q-H_{i},M,E\right)\text{,}$$

and

(9) $$q_{\text{new},i}=\{\begin{array}{l} q_{i},\;\text{if}\;S_{i,1}<S_{i,2}\;and\;S_{i,1}<S_{i,3}\\ q_{i}+h,\;\text{if}\;S_{i,2}<S_{i,1}\;and\;S_{i,2}<S_{i,3}\\ q_{i}-h,\;\text{if}\;S_{i,3}<S_{i,1}\;and\;S_{i,3}<S_{i,2} \end{array}\text{,}$$

where $H\in R^{6\times 6}$is a symmetric matrix of step sizes (h is 0.5 mm or degree in the present study), and $H_{i}$indicates the ith column vector. $q_{\text{new},\text{i}}$ is the ith new position or angle.

## 3 Results

### 3.1 Study 1

The condition of the minimum total score was: (1) target edge length, 3 mm; (2) threshold of thickness of the soft tissue, 0–20 mm; and (3) minimum distance between markers, 10 mm (see Appendix B for details).

### 3.2 Study 2

**Figure 6:**
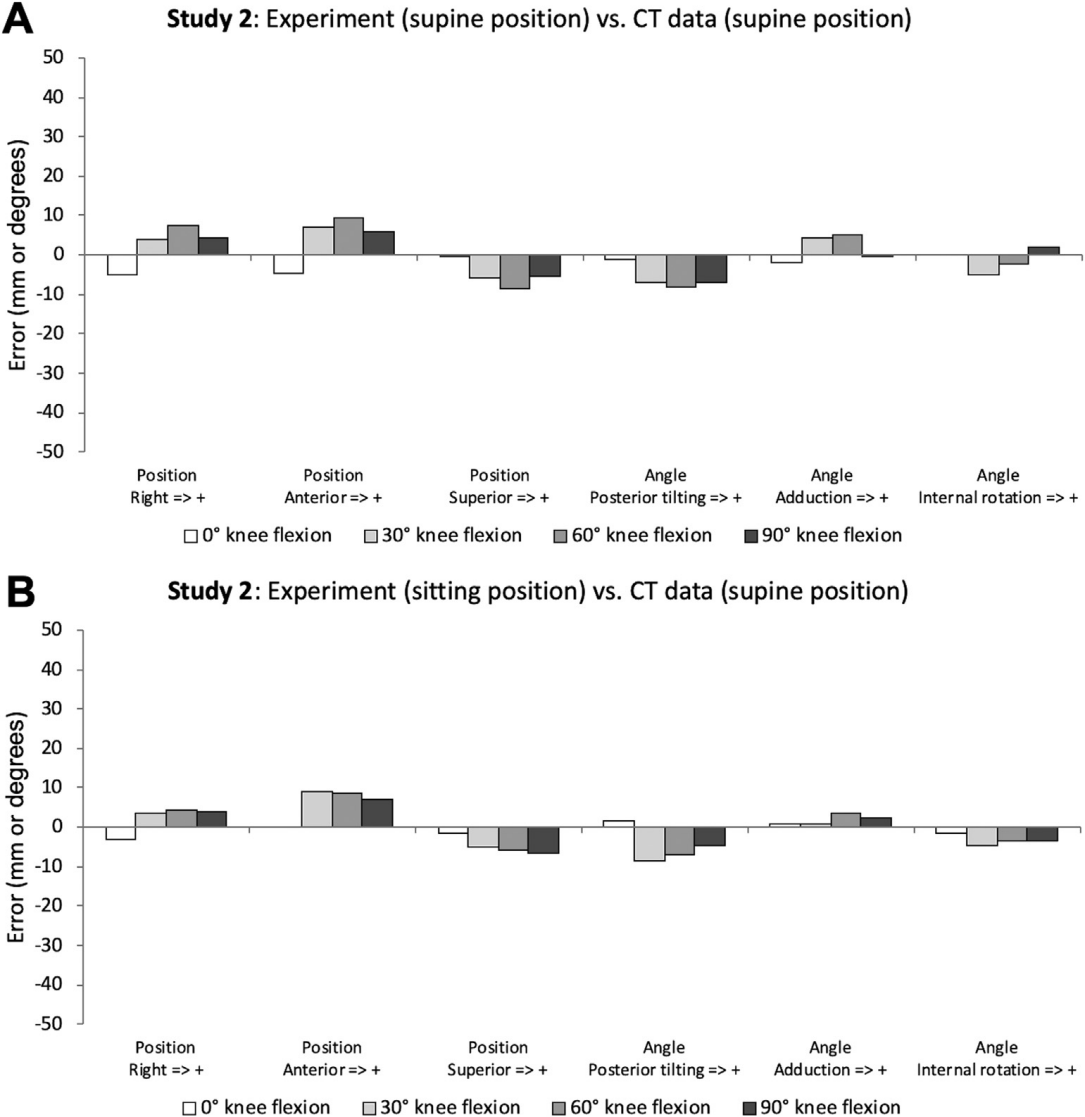
Effects of a difference in experimental posture (supine position or sitting position) on errors (i.e., positions and angles) between the true and estimated local coordinate systems (Study 2). A: Experiment (supine position) vs. CT data (supine position). B: Experiment (sitting position) vs. CT data (supine position). Both results show approximately similar errors despite the different postures.

Figure 6 shows the results of Study 2. In the supine position, the minimum errors of the positions and angles were 0.5 mm and 0.0°, respectively, with maximum errors of 9.5 mm and 8.0°, respectively. In the sitting position, the minimum errors of the positions and angles were 0.0 mm and 1.0°, respectively, with maximum errors of 8.5 mm and 8.5°, respectively. Based on these results, in the supine and sitting positions, the errors of the positions and angles were less than approximately 10 mm and 10° at most, respectively.

## 4 Discussion

The purposes of this study were (1) to explore and determine the most appropriate parameters for the CT-based surface mapping method (Study 1) and (2) to compare three-dimensional movement of the patella using the CT-based surface mapping method to that obtained from CT data (Study 2). Based on the results of Study 1, the most appropriate conditions were: (1) target edge length, 3 mm; (2) threshold of thickness of the soft tissue, 0–20 mm; and (3) minimum distance between markers, 10 mm. The results of Study 2 showed that, in the supine and sitting positions, the errors of the positions and angles were less than approximately 10 mm and 10° at most, respectively.

The novel point of the proposed method is that the positions and angles of the patella were examined using a CT-based surface mapping method with a motion capture system and CT without biplane fluoroscopy. The proposed method may be useful to evaluate three-dimensional movement of the patella under a static task. Furthermore, although three-dimensional movement of the patella was evaluated only under a static task, it may also be useful for a dynamic task with improvement. If an improved method for dynamic tasks is established in the future, the method can overcome some disadvantages of the 2D-3D registration technique: (1) shooting range is narrow; and (2) a dynamic task (e.g., running, cutting, and jumping) is limited by the fluoroscopy equipment (i.e., narrow experimental space).

Mattson et al. (2012) proposed a method, the surface mapping method, and evaluated three-dimensional movement of the scapula. However, the study generated many temporary solutions of positions and angles (6 degrees of freedom), and the sums of all vertical distances in each solution were calculated and evaluated. The algorithm can avoid the risk of catching a local minimum, but it has a high computational cost. On the other hand, a different algorithm using the modified steepest descent method was used to avoid the high computational cost. Therefore, although the computational cost was low (Study 2: mean (SD) 24.4 (3.0) s per trial), the risk of catching a local minimum should be evaluated in the future.

In Study 2, to examine the effect of posture on three-dimensional movement of the patella using the CT-based surface mapping method, two conditions (supine and sitting positions) were set in the experiment. However, the results of Study 2 (Figure 6) showed that the positions and angles of the patella were approximately the same even with different postures. Therefore, the effect of posture on three-dimensional movement of the patella appears small. Given this finding, it is possible to conduct an experiment in the sitting position with a Biodex system, ultrasonic echo, and electromyography. Thus, the proposed method may be useful to evaluate relationships between three-dimensional movement of the patella and various indices (e.g., knee extension strength, morphological features of the patellar tendon, and quadriceps muscle activity).

There are some limitations. First, whether the proposed method can be used to evaluate three-dimensional movement of the patella under weight-bearing movement (e.g., gait and running) is unknown. Therefore, the algorithm will be improved if needed, and it will be necessary to examine whether the improved CT-based surface mapping method can be used for evaluating weight-bearing movement. Second, the thickness of the soft tissue of the knee was calculated from CT data (i.e., static supine position without muscle contraction). However, muscle contraction (e.g., quadriceps contraction), even if the task is static, may change the thickness of the soft tissue of the knee. Therefore, this point should be examined in the future. Third, since the CT-based surface mapping method uses CT, there is a risk of radiation exposure. However, the risk is eliminated by using MRI, though it requires a long time for scanning. Fourth, the body mass index (BMI) of each subject of Studies 1 and 2 was 21.6 kg/m^2^ and 20.0 kg/m^2^, respectively. In cases of subjects with higher BMI values, the results of Studies 1 and 2 may differ. Therefore, the most appropriate parameters need to be examined, and three-dimensional movement of the patella should be evaluated in subjects with higher BMI values in a future study. Fifth, we were unable to resolve the relative movement between skin and bone. Errors of <10 mm and 10° are not small, and may have been caused by the relative movement between skin and bone. This problem is a limitation of the CT-based surface mapping method. Finally, although both knee joint angles on CT and the experiment were measured using a goniometer by the same experimenter, whether the angles were strictly the same is unknown. In Study 2, the true local coordinate systems of the patella were obtained from CT data for each condition (i.e., 0°, 30°, 60°, and 90° knee flexion). However, since knee flexion angles in the experiment with the motion capture system are not strictly the same compared to those obtained from CT data, the results of Figure 6 include experimental errors due to differences in knee flexion angles between CT data and the experiment. Therefore, a more accurate examination of the CT-based surface mapping method may be necessary.

A new method, CT-based surface mapping method, was proposed to evaluate three-dimensional movement of the patella. In Study 1, the most appropriate conditions were: (1) target edge length, 3 mm; (2) threshold of thickness of the soft tissue, 0–20 mm; and (3) minimum distance between markers, 10 mm. In Study 2, the errors of the positions and angles were less than approximately 10 mm and 10° at most, respectively (in both supine and sitting positions). A motion capture system and CT are available at many research institutes and hospitals. Although CT-based surface mapping may not be the most appropriate method for estimating three-dimensional movement of the patella, it could potentially serve as a fast and feasible method for estimating patellar movement in medical laboratory facilities. Therefore, CT-based surface mapping is most likely to be used in the research setting for the evaluation of three-dimensional movement of the patella as a static task. CT-based surface mapping (i.e., without fluoroscopy) may be useful in future studies for clarifying differences in three-dimensional movements of the patella between healthy subjects and patients with patellar tendinitis, between the two knees of the same subject, and between before and after surgery.

## Declarations

### Author contribution statement

Takuma Inai: Conceived and designed the experiments; Performed the experiments; Analyzed and interpreted the data; Contributed reagents, materials, analysis tools or data; Wrote the paper.

Tomoya Takabayashi, Masahiro Ikezu, Fumiya Kaneko, Kanta Matsuzawa & Mutsuaki Edama: Conceived and designed the experiments; Performed the experiments; Wrote the paper.

Satoshi Watanabe: Conceived and designed the experiments; Wrote the paper.

### Funding statement

This research did not receive any specific grant from funding agencies in the public, commercial, or not-for-profit sectors.

### Competing interest statement

The authors declare no conflict of interest.

### Additional information

No additional information is available for this paper.
