# Supplementary material for: A new method for estimating three-dimensional movement of the patella using a surface mapping method and computed tomography
Source: Heliyon. 2020 Aug 18;6(8):e04729. doi: 10.1016/j.heliyon.2020.e04729 (PMC7452489; doi:10.1016/j.heliyon.2020.e04729)
Supplement: Appendix A [file mmc1.docx]

**
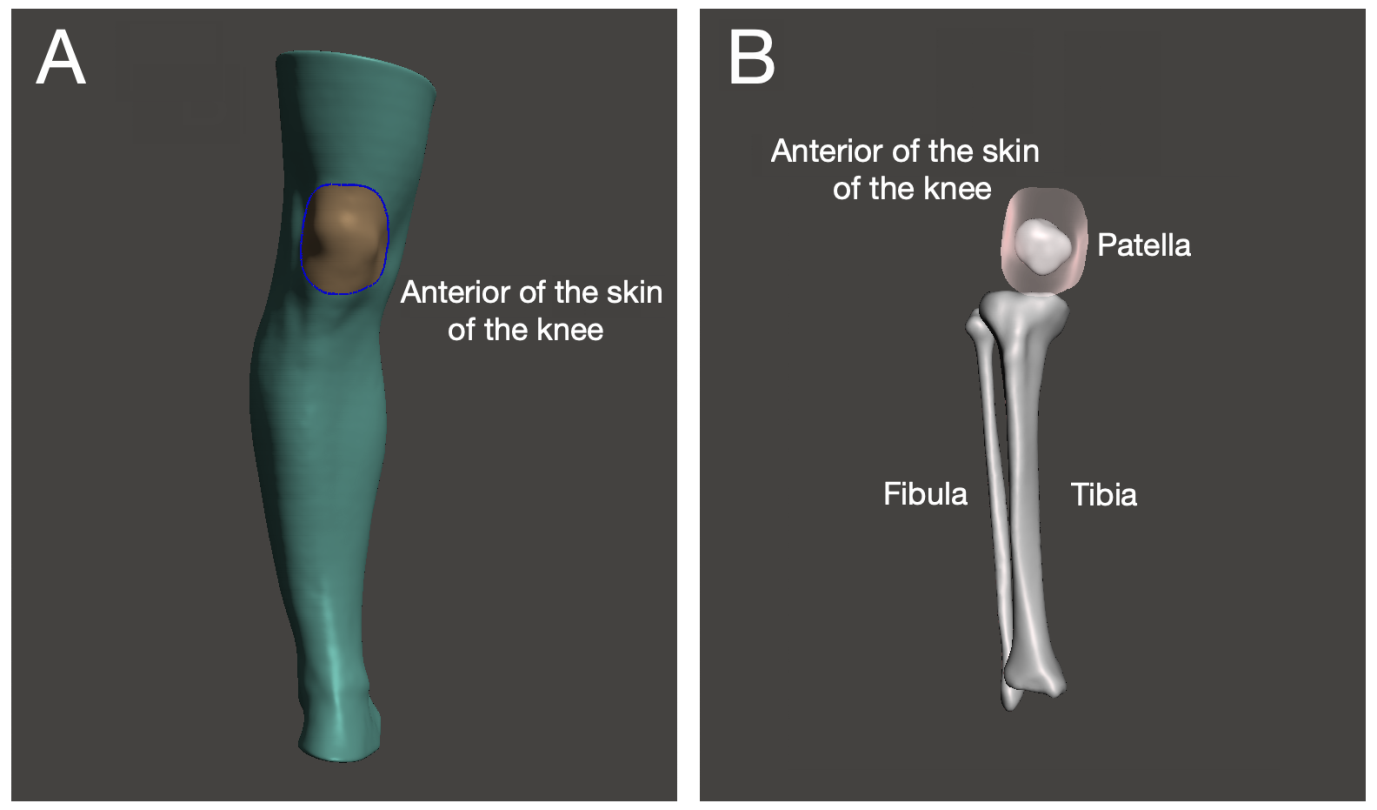
**

**Figure A1**. Three-dimensional model

A: Three-dimensional model of the skin of the right lower limb. Skin color indicates anterior of the skin of the knee. B: Three-dimensional model of the patella, tibia, and fibula of the right lower limb.

**Study 1**

**Creation of the three-dimensional model**

First, DICOM data for each condition were imported using open-source digital measurement software (Image J, NIH, Bethesda, MD, USA). Then, the image threshold was adjusted to extract the bone segment, and segmentation of the patella, tibia, and fibula was conducted. Similar to this process, the image threshold was adjusted to extract the skin of the right lower limb, and segmentation of the skin was conducted.

Three-dimensional models (patella, tibia, fibula, and skin of the lower limb) were exported as stl files. Next, the stl file of the skin of the right lower limb was imported to state-of-the-art software for working with triangle meshes (Meshmixer, Autodesk, Inc., CA, USA), and a smoothing process was conducted (Smoothing 2, Smooth scale 5, Constraint ring 3). Moreover, the anterior skin of the knee joint was trimmed from the right lower limb (Figure A1A). Similar to this process, the stl files of the patella, tibia, and fibula were also smoothed (Figure A1B).

**Definition of the local coordinate system of the patella**

Using a three-dimensional patella model obtained from CT data, the x-z plane of the patella was defined using principal component analysis (i.e., the y axis is the normal unit vector in the plane). Another plane including the y axis of the patella and coordinate point of the most inferior pole of the patella was calculated, and the x axis (plus is lateral) was defined as the normal unit vector in the y-z plane. The z axis was then decided using the cross product of the x and y axes. This local coordinate system is the true local coordinate system of the patella.

**Study 2**

**Creation of three-dimensional models**

Three-dimensional models (patella, tibia, fibula, and anterior of the skin of the knee) were created using the same procedure as in Study 1.

**Definition of the local coordinate system of the patella and lower leg**

The local coordinate system of the patella obtained from the CT data was created using the same procedure as in Study 1.

Two local coordinate systems of the lower leg were created by CT and the experiment (i.e., the motion capture system). The origin was set to the midpoint of lateral malleolus and medial malleolus. The z axis was defined as a unit vector from the lateral malleolus to the head of the right fibula. A plane including the z axis of the lower leg and the medial malleolus was calculated, and the y axis (plus is forward) was defined as the normal unit vector in the x-z plane. The x axis was then decided using the cross product of the y and z axes.
