# Supplementary material for: A new method for estimating three-dimensional movement of the patella using a surface mapping method and computed tomography
Source: Heliyon. 2020 Aug 18;6(8):e04729. doi: 10.1016/j.heliyon.2020.e04729 (PMC7452489; doi:10.1016/j.heliyon.2020.e04729)
Supplement: Supplementary file 2 [file mmc2.docx]

Appendix B

Table B1. Total score in each condition (Study 1)

Condition			
Target edge length [mm]	Thickness of the soft tissue [mm]	Minimum virtual marker distance [mm]	Total score
2	0–5	10	312
2	0–5	15	344
2	0–5	20	439
2	0–12.5	10	245
2	0–12.5	15	326
2	0–12.5	20	375
2	0–20	10	257
2	0–20	15	273
2	0–20	20	327
2	0–5	10	394
3	0–5	15	397
3	0–5	20	470
3	0–12.5	10	297
3	0–12.5	15	348
3	0–12.5	20	314
**3**	**0–20**	**10**	**235**
3	0–20	15	269
3	0–20	20	261
4	0–5	10	374
4	0–5	15	410
4	0–5	20	384
4	0–12.5	10	347
4	0–12.5	15	409
4	0–12.5	20	353
4	0–20	10	251
4	0–20	15	331
4	0–20	20	330

The gray high light indicates the most appropriate condition.

Appendix B

Table B2. Scores in each condition (Study 1)

Condition			Knee joint angle [deg]																								
			0						30						60						90						
			Position			Angle			Position			Angle			Position			Angle			Position			Angle			
Target edge length [mm]	Thickness of the soft tissue [mm]	Minimum virtual marker distance [mm]	x	y	z	x	y	z	x	y	z	x	y	z	x	y	z	x	y	z	x	y	z	x	y	z	Total score
2	0–5	10	4	7	6	1	6	13	6	21	16	27	17	26	26	12	4	6	13	10	18	20	19	2	6	26	312
2	0–5	15	14	8	12	14	19	12	2	27	6	13	6	9	15	13	19	16	19	21	4	15	10	22	23	25	344
2	0–5	20	23	17	22	24	25	10	18	26	9	26	5	25	4	23	15	14	14	24	22	23	12	25	25	8	439
2	0–12.5	10	5	3	3	5	2	19	25	6	8	17	25	14	16	2	10	3	17	15	7	8	18	4	3	10	245
2	0–12.5	15	15	10	10	4	14	21	16	11	13	25	24	16	10	7	20	12	20	6	25	4	7	9	13	14	326
2	0–12.5	20	17	13	25	13	16	24	23	10	15	19	20	18	27	9	6	11	16	11	8	14	11	19	8	22	375
2	0–20	10	16	4	1	3	1	25	13	5	19	18	18	15	18	1	11	2	15	5	13	11	14	7	10	12	257
2	0–20	15	7	6	5	7	22	14	26	13	27	11	9	5	23	6	14	13	24	7	10	6	5	3	4	6	273
2	0–20	20	19	12	9	9	26	27	11	14	18	22	3	8	22	11	16	10	8	3	24	10	25	5	2	13	327
2	0–5	10	8	9	8	2	20	20	1	22	22	23	27	21	12	25	2	26	12	8	16	26	16	24	26	18	394
3	0–5	15	11	15	16	12	27	15	15	25	7	20	1	22	17	21	26	22	1	13	21	22	22	26	5	15	397
3	0–5	20	25	18	18	18	18	11	10	24	21	21	23	27	25	27	12	25	4	26	27	27	2	14	27	20	470
3	0–12.5	10	1	2	4	8	7	16	22	1	4	7	22	13	24	8	23	7	23	16	14	21	17	21	15	1	297
3	0–12.5	15	13	11	14	16	15	17	27	8	24	15	4	12	11	17	1	5	26	19	26	17	4	18	24	4	348
3	0–12.5	20	10	16	20	22	24	26	19	15	2	5	14	17	13	10	5	9	25	17	15	2	1	1	7	19	314
**3**	**0–20**	**10**	**2**	**1**	**2**	**6**	**4**	**18**	**12**	**3**	**5**	**3**	**8**	**4**	**20**	**3**	**21**	**8**	**22**	**14**	**6**	**18**	**15**	**16**	**22**	**2**	**235**
3	0–20	15	6	5	7	23	9	22	17	7	11	4	12	2	14	4	9	1	9	1	19	24	27	12	21	3	269
3	0–20	20	21	14	11	10	21	23	9	12	17	1	13	6	1	5	3	4	10	2	5	7	20	23	12	11	261
4	0–5	10	20	27	21	20	5	8	7	20	23	24	11	24	8	22	13	19	11	18	1	16	13	11	16	16	374
4	0–5	15	26	19	23	21	12	7	24	18	1	14	2	23	7	18	27	23	2	20	17	25	9	27	18	27	410
4	0–5	20	24	26	27	27	8	9	8	23	10	12	10	7	9	26	18	21	7	9	23	19	23	20	9	9	384
4	0–12.5	10	9	21	19	11	10	3	20	4	25	9	21	3	19	20	25	24	21	4	20	3	21	10	1	24	347
4	0–12.5	15	18	25	24	25	11	5	5	16	26	16	26	11	5	24	22	20	18	27	9	9	24	8	14	21	409
4	0–12.5	20	27	22	26	15	13	6	21	17	3	6	7	10	21	16	8	27	5	12	11	13	26	17	17	7	353
4	0–20	10	3	20	17	17	3	1	14	2	12	2	15	1	6	15	24	17	6	22	12	5	6	15	11	5	251
4	0–20	15	12	23	15	26	17	2	3	19	14	8	16	19	2	19	7	18	27	25	2	1	8	6	19	23	331
4	0–20	20	22	24	13	19	23	4	4	9	20	10	19	20	3	14	17	15	3	23	3	12	3	13	20	17	330

The gray high light indicates the most appropriate condition.

Appendix B

Table B3. Average errors of the positions and angles in each condition (Study 1)

Condition			Knee joint angle [deg]																							
			0						30						60						90					
			Position [mm]			Angle [deg]			Position [mm]			Angle [deg]			Position [mm]			Angle [deg]			Position [mm]			Angle [deg]		
Target edge length [mm]	Thickness of the soft tissue [mm]	Minimum virtual marker distance [mm]	x	y	z	x	y	z	x	y	z	x	y	z	x	y	z	x	y	z	x	y	z	x	y	z
2	0–5	10	1.6	1.3	3.7	1.8	3.5	5.2	4.2	3.1	5.0	2.7	6.6	2.7	5.3	2.9	4.3	0.8	5.9	2.7	4.9	4.6	4.2	0.8	4.3	3.6
2	0–5	15	2.1	1.4	4.5	2.9	4.7	5.2	3.9	3.7	4.7	1.8	6.1	2.2	4.9	3.1	5.2	1.1	6.1	2.9	4.5	4.3	3.8	1.1	4.6	3.5
2	0–5	20	2.4	2.2	6.4	3.4	5.7	4.9	4.7	3.5	4.7	2.6	6.0	2.5	4.2	4.0	5.1	1.0	5.9	3.1	5.1	5.1	3.9	1.2	4.6	3.1
2	0–12.5	10	1.7	1.0	2.9	2.1	3.0	5.7	4.9	1.8	4.7	2.0	6.7	2.2	4.9	2.1	4.7	0.7	6.0	2.7	4.5	3.6	4.1	0.9	4.1	3.2
2	0–12.5	15	2.2	1.4	4.1	2.0	4.4	5.9	4.6	2.2	4.9	2.3	6.7	2.3	4.6	2.3	5.4	0.9	6.1	2.6	5.3	3.4	3.7	0.9	4.3	3.3
2	0–12.5	20	2.3	1.6	6.5	2.8	4.5	6.5	4.8	2.2	5.0	2.1	6.7	2.3	5.5	2.5	4.5	0.9	6.0	2.7	4.5	4.0	3.8	1.0	4.3	3.4
2	0–20	10	2.2	1.0	2.8	1.9	2.9	6.6	4.5	1.8	5.1	2.0	6.6	2.2	4.9	2.0	4.8	0.7	5.9	2.5	4.7	3.7	4.0	0.9	4.3	3.2
2	0–20	15	1.9	1.1	3.3	2.4	5.0	5.2	5.1	2.2	5.8	1.8	6.2	2.1	5.1	2.3	4.9	0.9	6.3	2.6	4.6	3.5	3.6	0.8	4.2	3.1
2	0–20	20	2.3	1.6	4.1	2.7	5.8	7.1	4.4	2.2	5.1	2.1	6.0	2.2	5.1	2.8	5.1	0.8	5.6	2.5	5.3	3.7	4.4	0.9	4.1	3.2
2	0–5	10	1.9	1.4	4.0	1.9	4.7	5.9	3.7	3.2	5.3	2.1	7.0	2.4	4.6	4.4	4.1	1.5	5.9	2.6	4.8	5.2	4.0	1.1	4.6	3.3
3	0–5	15	2.0	1.8	5.5	2.8	5.8	5.3	4.5	3.4	4.7	2.1	5.8	2.4	4.9	3.6	5.9	1.3	5.1	2.7	5.1	4.9	4.3	1.2	4.2	3.3
3	0–5	20	2.6	2.3	5.7	3.0	4.6	5.0	4.4	3.3	5.2	2.1	6.7	2.8	5.2	5.1	4.8	1.5	5.4	3.2	5.6	5.5	3.2	0.9	5.0	3.4
3	0–12.5	10	1.3	0.8	3.1	2.5	3.6	5.3	4.7	1.3	4.6	1.6	6.7	2.2	5.2	2.4	5.5	0.8	6.3	2.7	4.7	4.7	4.0	1.1	4.4	3.0
3	0–12.5	15	2.1	1.5	5.2	3.0	4.4	5.3	5.4	1.9	5.4	1.9	6.0	2.2	4.6	3.5	4.1	0.8	6.4	2.7	5.3	4.5	3.6	1.0	4.6	3.0
3	0–12.5	20	2.0	2.1	6.1	3.3	5.5	6.9	4.7	2.3	4.5	1.6	6.5	2.3	4.8	2.8	4.3	0.8	6.4	2.7	4.7	3.1	3.0	0.7	4.3	3.3
**3**	**0–20**	**10**	**1.4**	**0.7**	**2.9**	**2.4**	**3.4**	**5.6**	**4.5**	**1.5**	**4.6**	**1.4**	**6.2**	**2.1**	**5.1**	**2.1**	**5.4**	**0.8**	**6.2**	**2.7**	**4.5**	**4.5**	**4.0**	**1.0**	**4.6**	**3.0**
3	0–20	15	1.8	1.1	3.8	3.3	4.0	6.0	4.6	1.9	4.8	1.4	6.3	2.0	4.8	2.1	4.6	0.7	5.7	2.4	5.0	5.1	4.5	0.9	4.5	3.0
3	0–20	20	2.4	1.7	4.2	2.7	4.8	6.4	4.4	2.2	5.0	1.2	6.4	2.1	4.2	2.3	4.2	0.8	5.7	2.5	4.5	3.5	4.3	1.1	4.3	3.2
4	0–5	10	2.4	3.9	6.3	3.1	3.5	4.4	4.3	3.0	5.3	2.2	6.3	2.5	4.4	3.9	4.9	1.3	5.7	2.7	4.2	4.4	3.9	0.9	4.4	3.3
4	0–5	15	2.6	3.4	6.4	3.2	4.1	4.2	4.8	2.8	4.2	1.8	5.9	2.5	4.4	3.5	5.9	1.3	5.2	2.8	4.8	5.1	3.8	1.5	4.5	3.7
4	0–5	20	2.6	3.7	6.9	3.8	3.8	4.4	4.4	3.3	4.8	1.8	6.2	2.1	4.6	5.0	5.2	1.3	5.6	2.6	5.2	4.5	4.4	1.0	4.3	3.1
4	0–12.5	10	2.0	3.5	6.0	2.8	4.0	3.5	4.7	1.6	5.5	1.7	6.7	2.0	5.0	3.6	5.9	1.3	6.1	2.5	5.0	3.3	4.3	0.9	4.1	3.4
4	0–12.5	15	2.3	3.6	6.5	3.6	4.1	4.0	4.2	2.4	5.7	1.9	6.9	2.2	4.3	4.1	5.4	1.3	6.0	3.2	4.5	3.6	4.4	0.9	4.4	3.4
4	0–12.5	20	2.9	3.5	6.5	2.9	4.2	4.2	4.7	2.7	4.6	1.6	6.1	2.2	5.1	3.4	4.6	1.6	5.5	2.7	4.6	3.9	4.5	1.0	4.4	3.1
4	0–20	10	1.6	3.5	5.5	3.0	3.2	3.2	4.5	1.5	4.8	1.3	6.6	1.9	4.3	3.3	5.5	1.2	5.6	3.0	4.7	3.4	3.7	1.0	4.3	3.1
4	0–20	15	2.0	3.6	5.3	3.6	4.6	3.2	3.9	2.9	5.0	1.6	6.6	2.4	4.2	3.5	4.5	1.2	6.5	3.1	4.4	3.1	3.7	0.9	4.5	3.4
4	0–20	20	2.4	3.6	5.1	3.0	5.3	3.8	4.0	2.1	5.1	1.7	6.6	2.4	4.2	3.1	5.2	1.1	5.4	3.0	4.4	3.8	3.4	0.9	4.5	3.3

The gray high light indicates the most appropriate condition.
